# Simulative validation of a novel experiment carrier for the Einstein-Elevator

**DOI:** 10.1038/s41598-023-46483-4

**Published:** 2023-11-08

**Authors:** Richard Sperling, Marvin Raupert, Christoph Lotz, Ludger Overmeyer

**Affiliations:** 1 Institut für Transport- und Automatisierungstechnik, Leibniz Universität Hannover, An der Universität 2, 30823 Garbsen, Germany; 2 Institut für Satellitengeodäsie und Inertialsensorik, Deutsches Zentrum für Luft- und Raumfahrt e. V. (DLR), Callinstr. 36, 30167 Hannover, Germany

**Keywords:** Mechanical engineering, Aerospace engineering

## Abstract

In order to develop hardware that can be used in space, tests under those space conditions are often important to ensure the functionality in advance. Facilities that are used to recreate gravity conditions of space include space stations, satellites, parabolic flights and earthbound facilities. Drop towers are earthbound facilities, that can replicate the gravitational conditions of free falling in space by dropping objects. Those objects would not experience any measurable force due to gravity according to Einstein’s famous thought experiment. The Einstein-Elevator is one of the first active driven drop towers with an experiment carrier falling inside a gondola. A major indicator for the quality of the facility is the residual acceleration of the payload. With the Einstein-Elevators current setup vibrations of the experiment carrier cause measurable residual accelerations of higher than $10^{-3}$*g*. To achieve the targeted 0-*g*-quality with a residual acceleration of less than 1 $\mu$*g* (microgravity) in the Einstein-Elevator, a new experiment carrier is required that minimizes the residual acceleration for a payload. This paper describes a design of the experiment carrier for the Einstein-Elevator that is able to reach microgravity and analyzes its functionality using FEM-simulations.

## Introduction

To cope with the rising demand for research under space conditions such as microgravity, research facilities for those conditions are beeing built^1,2^. Currently, there are different types of facilities that can create a $\mu$*g*-environment. Space stations^3^, satellites^4^, rocket missions^5,6^, parabolic flights^7^ and earthbound facilities including drop towers like the Einstein-Elevator are currently used for research under $\mu$*g*^8–13^. Some of those facilities are even able to replicate partial gravity in the range of 0 to 1 *g*. To compare those facilities in terms of $\mu$*g*-quality, the residual acceleration is commonly measured. In general, lower residual accelerations yield higher quality results as any disturbance reduces the reproducibility of the experiment. However, some experiments especially those performed under Moon-gravity or Mars-gravity such as^14^ do not have high requirements on the residual acceleration as small vibrations are overshadowed by the gravitational fluctuation of the celestial body itself. When relatively high gravity is required for the experiment small vibrations do not have a big impact on the results as they are overshadowed. Nevertheless, experiments in the field of physics often have high requirements on the environment. Those requirements can include a low magnetic field, a low electric field, a high vacuum quality and low residual acceleration. Improving the quality of the microgravity ($\mu$*g*) in terms of residual acceleration can be achieved by decoupling sources of vibration from the payload. In case of the Einstein-Elevator those sources of vibration are the linear motors of the gondola, the guidance system or even ambient sound outside the gondola. To create a microgravity environment those sources need to be decoupled from the experiment carrier inside the gondola that houses the payload. Currently a experiment carrier is beeing used that is strongly affected by those disturbances. To remove remaining vibrations after decoupling an experiment carrier is required that does not pick up on those vibrations or is able to dampen them quickly^8^.

The experiment carrier is currently being built and will be integrated into the Einstein-Elevator. In future work, this carrier will used for various planned experiments in physics such as atom interferometry^15,16^. Those experiments are sensitive to vibrations and therefore require low residual acceleration below 1 $\mu$*g*. Also, experiments in the field of engineering are planned. This includes, for instance, laser-based additive manufacturing on the moon and Mars using regolith^14,17^. In another project, the laser metal deposition process is being researched in microgravity (DFG project number: 456663377).

This paper analyzes a specific carrier design to enable microgravity for experiments in the Einstein-Elevator using finite element analysis. The first step was to analyze the behavior of the carrier design under a static load. To get results that are closer to the real behavior of the carrier during operation the carrier is analyzed under a dynamic load that could be measured in the Einstein-Elevator. The goal is to determine the residual acceleration for further improvements of the $\mu$*g*-quality.

## Design of the Einstein-Elevator

The Einstein-Elevator is an active driven drop tower allowing to perform experiments under microgravity conditions for up to 4 s. Higher adjustable gravitational conditions (hypogravity) can even be maintained for a longer duration, because in those cases the drives would counteract Earth’ acceleration. Inside the towers gondola, an experiment carrier is placed that can hold a payload with a diameter of up to 1.7 m, a height of 2.0 m. The mass of carrier and payload can reach up to 1000 kg. A vacuum can be created inside the gondola for acoustic decoupling. This is required as the noise of the facility otherwise prevents the experiment carrier to reach residual accelerations below $10^{-3}$*g*. With this setup, a repetition rate of 300 experiments per day can be reached in a three-shift operation^11,18^.

The Einstein-Elevator is designed to minimize any sources of vibrations. For example, the basic structure consists of two tower support structures standing completely separated one inside the other. The inner supporting structure holds the drive and serves to guide the drive carts. For minimal interference, only the guidance of the gondola is mounted on the outer supporting structure. The two towers are connected only by the coupling rod, which connects the gondola to the two drive carts via the traverse. This design prevents the transfer of horizontal disturbances into the experiment and only transmits the vertical feed force^11,18^. To avoid cables for power and data connection the system buffers energy in batteries and provides optical data couplers.

**Figure 1:**
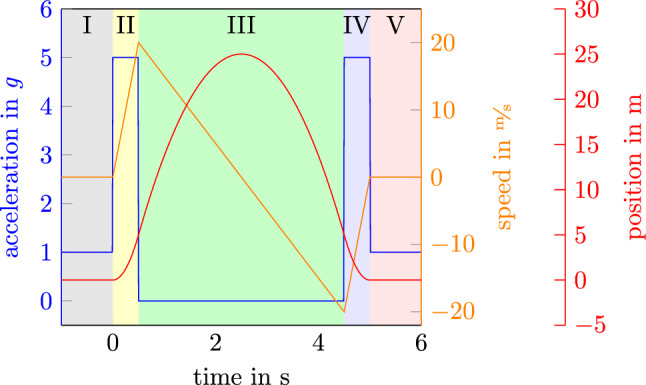
Phases during a microgravity experiment in the Einstein-Elevator with their ideal profile(I: Pre-Launch-Phase, II: Launch-Phase, III: Free-Fall-Phase, IV: Decceleration-Phase, V: Post-Launch-Phase).

Different gravity profiles can be reproduced within the system. However, this paper focuses on the parabolic microgravity flight. A usual flight for a microgravity experiment can be split into 5 phases as shown in Fig. 1.

I *Pre-Launch-Phase* The preparation phase is at the beginning of each flight. During this phase, the experiment is set up or calibrated and the facility is prepared for launch. This includes evacuating the gondola to acoustically decouple the experiment carrier from the surrounding facility and centering the experiment carrier in the center of the gondola. This phase ends by launching the gondola.
II *Launch-Phase* The second phase is the acceleration phase. During this phase, the gondola is accelerated upwards with 4 *g* for about half a second. Combined with the gravitation of the Earth 5 *g* can be recorded at the gondola and the experiment carrier.
III *Free-Fall-Phase* The third phase is the flight-phase, microgravity-phase or free-fall-phase. After acceleration, the gondola detaches from the carrier by deccelerating it for a fraction of a second and allowing the inertia of the carrier to lift the carrier of the gondola floor. Then, the Carrier has no physical connection to the surrounding structure. During this period the gondola is controlled to keep a defined distance to the carrier. The carrier travels in a vertical parabola up and down. Within this time the carrier is free-falling and the acceleration is ideally less than 1 $\mu$*g* while the experiment is conducted. The free-fall-phase lasts up to 4 s.
IV *Decceleration-Phase* Before the gondola stops at the parking position, it is brought closer to the carrier so that they decelerate together to a full stop. Similar to the acceleration phase high accelerations will be measured.
V *Post-Launch-Phase* The post-launch phase is used to secure the experiment data and to re-center the experiment carrier. In experiment campaigns in which several flights are carried out in succession, work steps of the pre- and post-launch phases are carried out simultaneously.

Througout those phases, the acceleration of the gondola and the experiment carrier is measured with multiple sensors. The analog sensor data is converted to digital values using a ADC with a resolution of 24 bit (Model: ELM3004-0000, Company: Beckhoff). A K-Beam MEMS accelerometer with a range of ± 50 *g* (Model: 8396A050, Company: Kistler) is used to measure the acceleration during the launch and the decceleration. This sensor combined with the ADC allows the measurements to have a resolution of 7.5 $\mu$*g*. To measure the residual accelerations close to 0 *g* during the free-fall a Titan accelerometer (Model: TACCL-N1, Company: Nanometrics) is used. This sensor allows the measurements to have a resolution of 50 n*g* to 700 n*g* depending on the setting. However, this sensor is not able to measure the acceleration during the launch as it exceeds the measurement range of the sensor.

One of the biggest challenges of reaching microgravity is the excitation of the facility’s components during the acceleration phase. Despite its design not all sources of vibrations can be decoupled. Vibrations caused by the linear drive during and its guidance as well as the guidance of the gondola can be transferred to the experiment carrier during the launch-Phase. Reducing the impact of those vibrations can be achieved by increasing the stiffness of carrier. With this the carrier would be less susceptible to low excitation frequencies. Another would be to dissiplate the vibrational energy with active or passive damping during the free-fall-phase.

## Design of the experiment carrier

**Figure 2:**
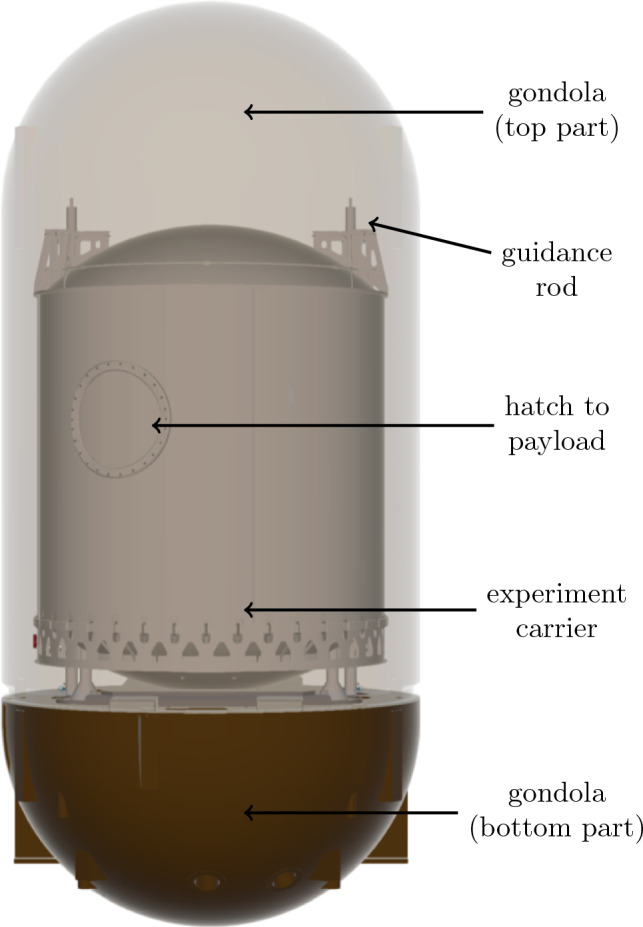
Experiment carrier inside the evacuated gondola; gondola top part is shown transparently.

To carry out experiments payloads have to be secured to specific facility equipment. While facilities such as parabolic flights usually require payloads to be attached to the aircraft in order to be safe around humans, other microgravity facilities such as the GraviTower Bremen Prototype at the ZARM in Bremen^19,20^, the electromagnetic microgravity facility in Beijing^21^ and the Tsinghua University Freefall Facility^12^ use a free-flying experiment carrier within a capsule acting as a drag shield. Those facilities use a complex release mechanism to cut the physical connection between the experiment carrier and the surrounding capsule. The Einstein-Elevator also uses a design with a carrier within a gondola acting as the drag shield. However, the gondola can be controlled to compensate for the air drag and friction losses of the guides and to prevent contact between the experiment carrier and the gondola. Figure 2 shows the setup of the experiment carrier within the gondola.

**Figure 3:**
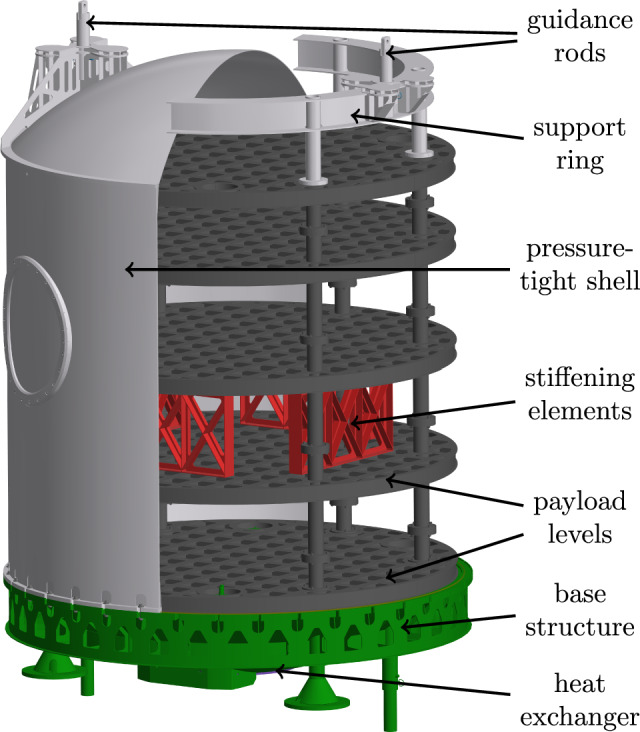
Experiment carrier; cross-section of the pressure tight shell and support ring; the pressure tight shell and the support ring are not used at the same time.

**Figure 4:**
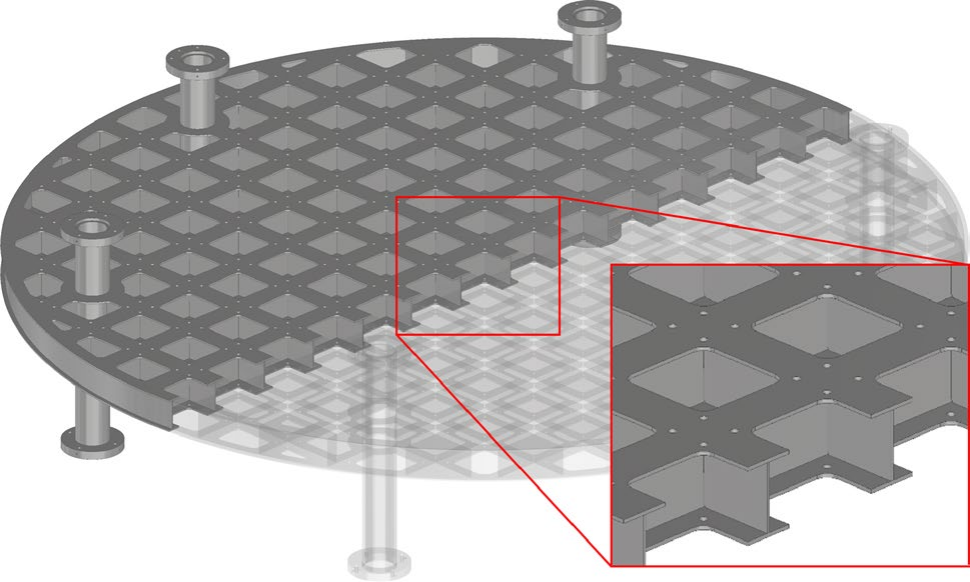
Construction of a payload level.

**Figure 5:**
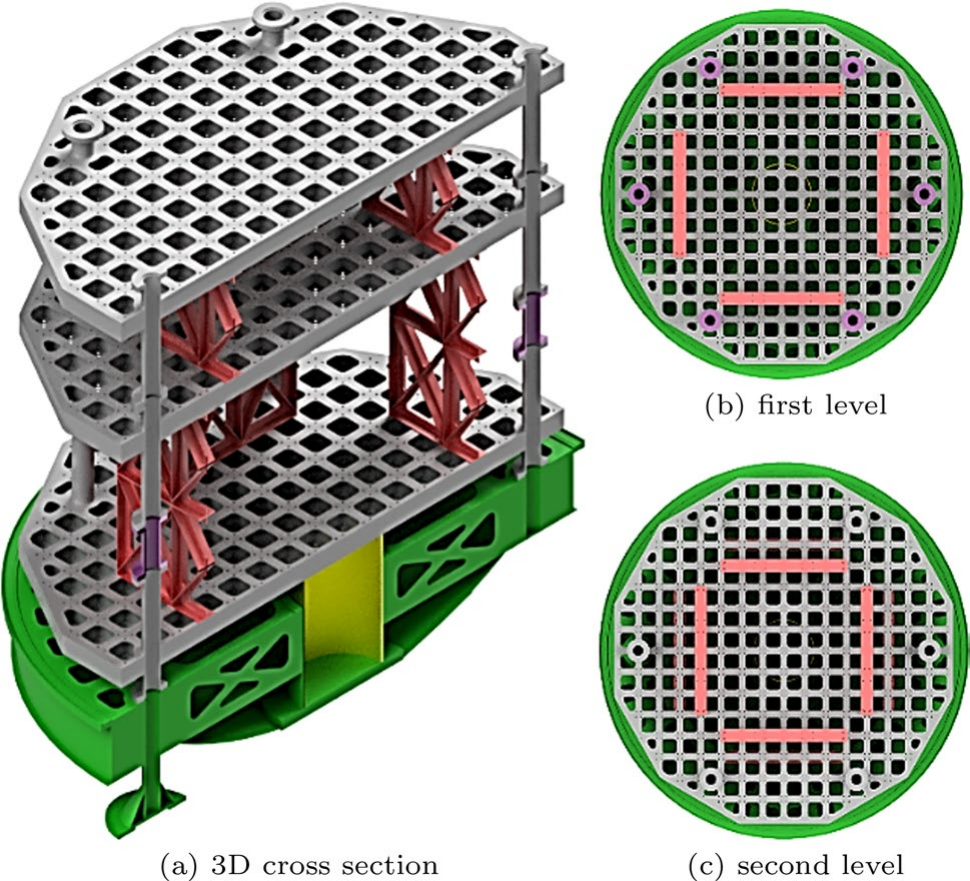
Example for a configuration of the experiment carrier.

**Figure 6:**
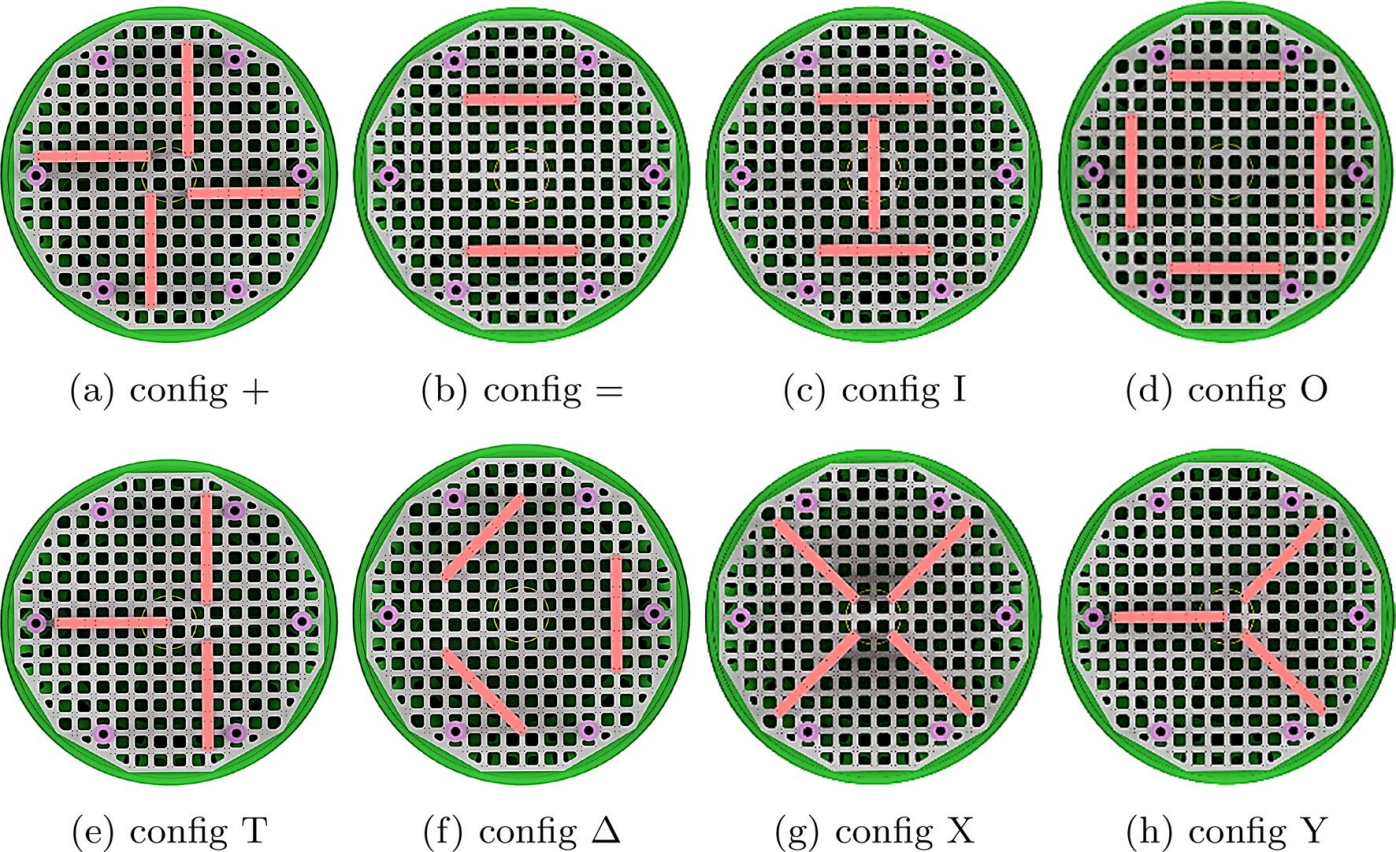
Other configurations considered in the analysis.

As a main goal, for achieving a minimum of residual acceleration during the $\mu$*g*-phase the carrier is designed as a highly stiff construction. Figure 3 shows the assembled experiment carrier. The carrier is built out of a base (green), which contains all the infrastructure hardware such as flight computer, inertial measurement unit, the power supply unit, the pump for the cooling system and the vacuum valve. All those components are placed inside their own pressure vessel to not be limited to vacuum compatible parts. A set of sockets that connect the hardware inside the pressure vessel are accessible for the payload. At those sockets cooling water, a 24 V power supply, a network connection as well as a variety of trigger signals at different voltage levels are available. The trigger signals can be used to indicate the start and end of each of the phases described previously. Centered at the bottom of the base is the heat exchanger. The heat exchanger is physically connected to its counterpart on the gondola’s floor between flights and during the acceleration phase. Above the base structure is space for the experiment hardware which can be enclosed by a pressure-tight shell (light gray cross-section) to separate the experiment space from the gondola’s vacuum. On top of the shell guidance rods are mounted. Those rods limit the translational and rotational movement of the carrier in case of malfunctions. A hatch allows quick but limited access to inside of the carrier without removing the shell. This pressure-tight shell is optional and can be removed if the experiment hardware is able to function in a vacuum or a vacuum inside the gondola is not required for acoustic decoupling. However, when operating the carrier without its pressure tight shell, a support ring (light gray cross-section) is required to hold the guidance rods. Inside the pressure tight shell and on the base structure, payloads can be mounted on modular expandable payload levels (dark gray). On these levels, there is a 12x12 cm mounting grid with 8x8 cm cutouts to reduce mass. Two aluminum plates are welded on both sides of vertically aligned plates with the 12x12 cm grid to create those payload levels, effectively creating many I-beam-shaped cross-sections (see Fig. 4). The levels are connected by 6 legs which are screwed together. All six legs have a length of 120 mm on the top of the mounting grid and a length of 240 mm on the bottom. For more flexibility, those levels can be flipped upside down to change the spacing between individual levels. Vertical forces can be transferred to the bottom of the carrier and into the gondola through those legs. Forces can also be transferred in the middle of the first payload level through the heat exchanger. Additional stiffening elements (red) increase the stiffness between two levels if it is necessary. Those can be oriented in 45° or 90° angles relative to the grid of the levels. For example, the levels above the first level can be reinforced in the center to utilize the support of the heat exchanger. However, as a trade-off, those stiffening elements split the payload volume into multiple sections. Instead of stiffening elements, the actual payload or parts of it could be secured between two levels to act in a similar way. Figure 5 shows an example of a possible configuration. It allows some stiffening while maintaining a majority of payload volume. Other configurations shown in Fig. 6 were analyzed in this work. Figure 6g allows for a stiffer carrier at the trade-off of splitting the payload volume into many small chunks. The maximum mass of the payload is dependent on the chosen configuration as the maximum total mass of 1000 kg includes the mass of the carrier itself. Table 1 shows the mass of each individual part. A minimum carrier mass of 230 kg can be reached leaving 770 kg for the payload. A configuration with 5 payload levels, 4 stiffening elements between individual levels and a pressure-tight shell would weigh 598 kg leaving 402 kg for the payload.

**Table 1:** Mass of individual carrier parts.

Part	Mass (kg)
Base (mandatory)	180
Pressure-tight shell (optional)	120
Payload level (1–5 mandatory)	50
Stiffening element (optional)	3

## Static analysis

**Figure 7:**
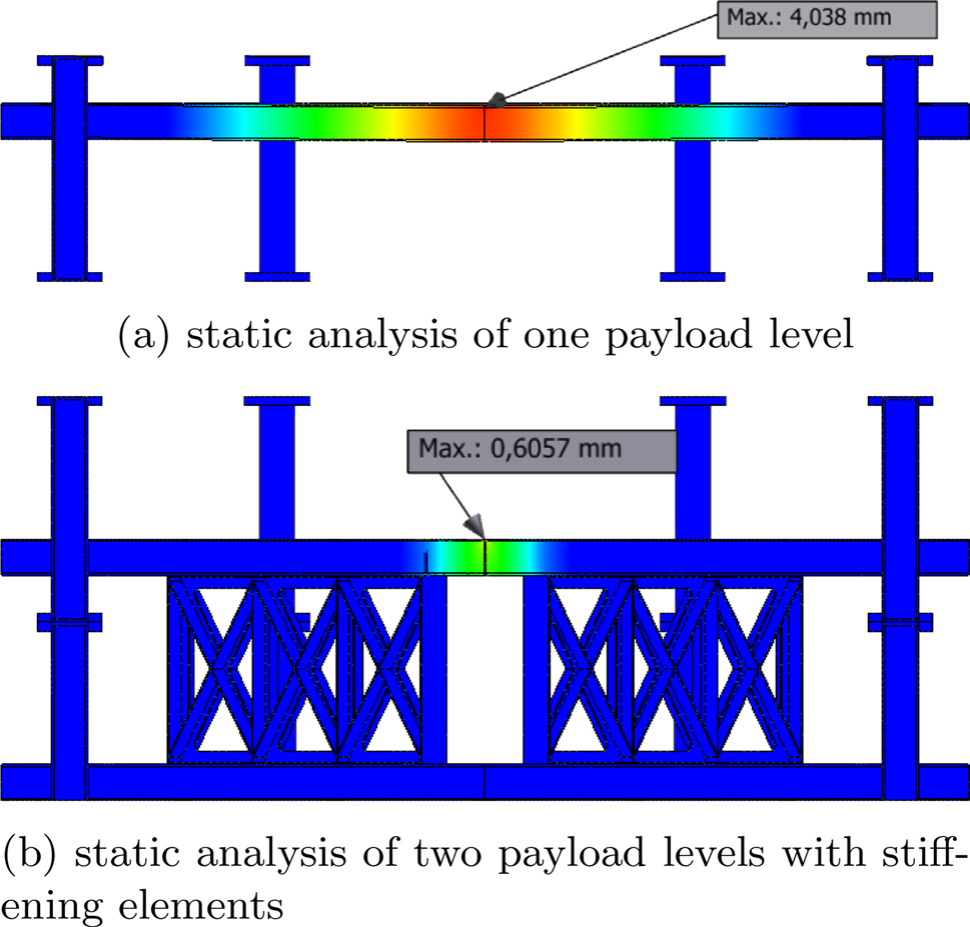
Static analysis of one payload level (**a**) and two payload levels with stiffening elements (**b**) with a point mass of 500 kg.

By determining the static behavior of the system it can be determined whether the carrier is able to withstand the load during the acceleration phase. The payload levels above the first level do not necessarily have support in the center and thus are the most sensitive parts of the construction. Therefore, only an analysis for the upper levels will be conducted to reduce computing time. Figure 7a shows the displacement of one payload level with a 500 kg payload as point mass in the center under an acceleration of 5 *g*. The maximum displacement for this scenario is about 4 mm. By adding stiffening elements in the center between two levels the displacements can be reduced significantly as shown in Fig. 7b. The maximum displacement, in this case, is reduced to about 0.6 mm. Reducing the displacement would also reduce the maximum amplitude of vibrations and thus increase the $\mu$*g*-quality. In general, supporting payload levels helps to transfer vertical forces through the feet and heat exchanger into the gondola and away from a sensitive payload. To achieve the maximum effect, the stiffening elements should be placed between locations with the most displacement and points where forces can be transferred to the gondola.

## Dynamic analysis

After the acceleration phase deformations will cause vibrations in the system. Especially vibrations at the payload will lead to unwanted residual acceleration. To reach microgravity quality below 1 $\mu$*g*, those vibrations have to be dampened within the first second of the free-fall phase to leave at least 3 s for the experiment. In general, the faster those vibrations are dampened, the longer the microgravity will last. Therefore, a further dynamic analysis of the carrier is needed.

The software used for the analysis is Ansys. A transient structural analysis was chosen as this type of analysis simulates time-dependent behavior of a system. As a finite element analysis, the structure is meshed into smaller elements and nodes. Equation (1) is the underlying differential equation for each of those nodes. The mass matrix *M*, the damping matrix *C* and the stiffness matrix *K* are derived from material properties and the shape of the elements. *F*(*t*) is the applied load vector and consists of outside forces or gravity. Within a simulation, Ansys solves this partial differential equation numerically for the nodal displacement *u*(*t*). The residual acceleration can be calculated by differentiating the displacement twice ($\ddot{u}(t)$).

1 $$\begin{aligned} M \ddot{u}(t) + C \dot{u}(t) + K u(t) = F(t) \end{aligned}$$

**Figure 8:**
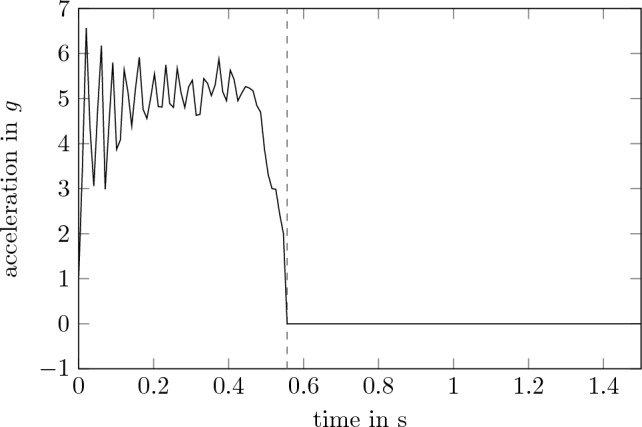
Input acceleration (launch-phase ends and $\mu$*g*-phase starts at 0.56 s).

The simulation is performed for different configurations of the carrier with differently positioned stiffening elements as described in Section “Design of the experiment carrier”. Each configuration is simulated under the same conditions. 150 steps with a step size of 0.01 s are performed. According to the Nyquist-Shannon sampling theorem loads with frequencies of up to 50 Hz can be replicated with this sampling time. Even though a normal $\mu$*g*-flight in the Einstein-Elevator can last for up to 4 s, only 1.5 s are simulated for a significant reduction of computation time. The first 560 ms correspond to the acceleration phase. It is assumed that the experiment carrier with its three feet as well as the heat exchanger is fixed to the bottom of the gondola. For the acceleration input during launch, the measured acceleration of a real flight is used (Fig. 8). As the gondola only exerts force on the experiment carrier in vertical direction, horizontal residual accelerations are assumed to be a result of acoustic noise. Therefore, horizontal accelerations are neglected in this analysis. This measured acceleration has a mean of 5 *g* and includes the vibrations that occur at the gondola. That way the carrier is stimulated with excitation frequencies of the real system. During this phase, the carrier is fixed at the feet and the heat exchanger. Afterwards, throughout the $\mu$*g*-phase, those restrictions are lifted to allow the carrier to float freely. Because no physical connection between the gondola and therefore the sources of vibrations, the acceleration applied to the carrier is constant at zero for the remaining simulated time. In this phase, the acceleration is not uniform across a single payload level. The acceleration is graphed at the point that has the most acceleration. This point varies depending on the configuration.

**Figure 9:**
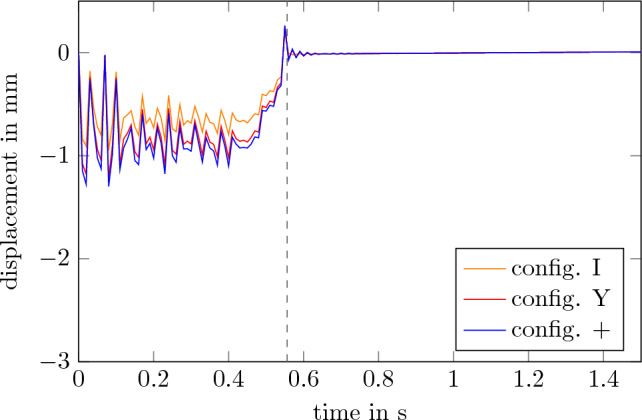
Displacement of the payload levels.

**Figure 10:**
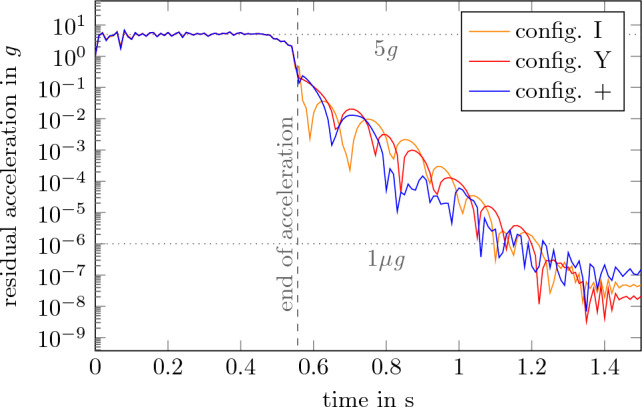
Absolute residual acceleration as a result of the deformation.

The results of those simulations are shown in Figs. 9 and 10. During the acceleration phase, the displacement and the residual acceleration correlates with the input gravity profile. After that, both values 0. Within less than 600 ms all configurations reach an residual acceleration of less than 1 $\mu$*g*. So it can be assumed that 3.4 s of microgravity can be achieved with those configurations of the final experiment carrier. Other configurations of the carrier yield similar results and are therefore not shown here. As described previously those results can only be applied to low frequencies of less than 50 Hz. As higher frequencies can still relate to structural vibrations it is usefull to furhter evaluate the behavior^22^.

**Figure 11:**
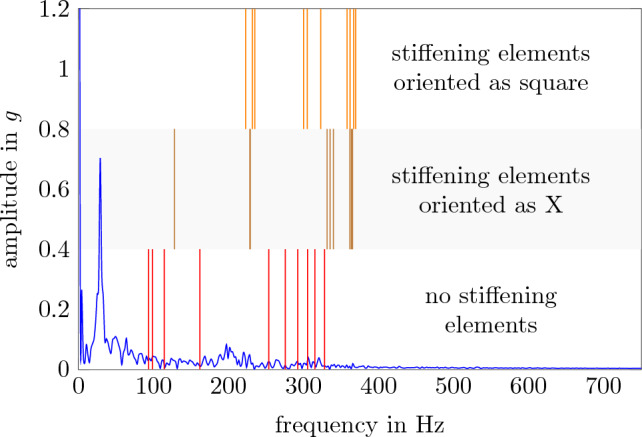
Frequency spectrum of the excitation during the acceleration phase and the first 10 eigenfrequencies of three different configurations.

In order to analyze the impact of higher frequency excitations, it is useful to understand what higher frequencies are important to analyze. The critical frequencies in this case are the eigenfrequencies of the system. Figure 11 shows the frequency spectrum of the excitation during the acceleration phase and the first 10 eigenfrequencies for different configurations of the payload levels with stiffening elements. The strongest excitation happens with frequencies less than 50 Hz. For higher frequencies, the amplitude is significantly lower. Due to the damping of the material itself high frequency accelerations have a smaller impact on the payload. So shifting the vibrations to higher frequencies would lead to lower residual accelerations at the payload. For a single payload level without stiffening elements, the first eigenfrequency is 93 Hz, which already is beyond the most dominant excitation frequencies. By adding stiffening elements those eigenfrequencies can be manipulated to be higher and thereby avoiding critical states.

Though the base and the pressure-tight shell are not directly connected to the payload, the eigenfrequencies are can still impact the payload. However those frequencies can hardly be changed. With 113 Hz for the base and 72 Hz for the pressure-tight shell, the eigenfrequencies are still high enough to avoid the most dominant excitation frequency.

## Summary

In the Einstein-Elevator experiments can be conducted under conditions such as microgravity. In order to do that, the facility’s carrier needs to be as stiff as possible to reduce the residual acceleration below 1 $\mu$*g*. The design for the experiment carrier was described. The presented design utilizes the capabilities of the Einstein-Elevator, such as the large payload volume and the vacuum within the gondola. Furthermore it allows active cooling of the carrier hardware. The design by itself already has high stiffness. It is able to dampen vibrations with less than 50 Hz caused by the components of the Einstein-Elevator quickly to reach microgravity within 0.6 s. Future work will include an analysis of the real experiment carrier. Due to the high flexibility of the carrier stiffening elements are needed to reach the same quality for any kind of payload. Using those stiffening elements the eigenfrequencies of the system can be tweaked and strong excitation frequencies of the facility can be avoided. It could be shown that despite the large size of the carrier, results can be achieved, similar to best $\mu$*g*-quality, that can be reached throughout other facilities.

As the carrier is built to be used with many different payloads, it is difficult to determine the optimal placement of the stiffening elements. Finding the optimal placement for stiffening elements would require a lot of time-consuming simulations Future work will include an optimization of the model to speed up the simulation. The faster model will then be implemented in a tool that can automatically find the best placement of stiffening elements to maximize the stiffness. This would improve the $\mu$*g*-quality. Another way to improve the quality is by increasing the dampening. This could be done by placing damping materials between payload levels. Active dampers can also be used to reduce vibrations. However, those dampers could have a negative impact on the stiffness of the system. Damping vibrations will be part of future work.

The experiment carrier system makes it possible to integrate complex experimental setups of any kind. One of the first projects will be the research on laser metal deposition in microgravity, where components with different sizes and masses have to be placed in a very specific arrangement over the entire height of the experiment carrier. This includes, for instance, laser equipment, processing optics, energy source, process chamber and handling systems. The flexible design makes it possible to distribute the setup over several levels while mounting the components with higher mass as low as possible. Sensitive components such as the processing optics and the laser can also be stabilized by using another level or the stiffening elements. Cables, optical fibres, tubes etc. for transmitting signals, power, light and fluids can easily be routed through the cutouts of the mounting grids. While a project is being executed, the construction of another project can be prepared in parallel. Therefore, additional identical levels are provided, which then only have to be exchanged in the experiment carrier.

## Data Availability

The datasets used and/or analysed during the current study is available from the corresponding author or the co-authors on reasonable request?
